# From HTA to the bedside: aligning GenAI validation with clinical reality

**DOI:** 10.1017/S026646232610378X

**Published:** 2026-04-17

**Authors:** Zekai Yu, Weihao Cheng, Feiwei Qin, Wei Xiong, Yang Hu

**Affiliations:** 1 School of Computer Science and Technology, Hangzhou Dianzi University, China; 2 School of Communication Engineering, Hangzhou Dianzi University, China; 3 Neonatal Intensive Care Unit (NICU), Department of Neonatology, Shangrao Maternal and Child Health Hospital, China; 4 Institute of Malaysia and International Studies (IKMAS), Universiti Kebangsaan Malaysia, Malaysia

We read with great interest the recent article by Fleurence and Chhatwal on implementing generative AI (GenAI) into Health Technology Assessment (HTA) practice (1). The authors present a highly relevant framework, emphasizing that the readiness of GenAI is heavily task-dependent and should serve to augment, rather than replace, human expertise in bounded tasks.

This perspective strongly resonates with current challenges in clinical big data mining and the deployment of bedside AI. The authors correctly point out that GenAI excels in well-defined tasks such as systematic literature review screening and structured data extraction, yet struggles with fully autonomous, end-to-end reasoning. In the broader intersection of AI and medicine, particularly when analyzing complex clinical datasets, this distinction is crucial. While GenAI can rapidly process unstructured clinical text, deriving actionable, causal insights requires rigorous methodological constraints and human-in-the-loop accountability.

Furthermore, the article’s focus on transparent reporting and task-level validation—echoing the ISPOR ELEVATE-GenAI guidelines (2)—is a necessary step toward building trust. In clinical applications, a model’s performance metrics are insufficient without clear documentation addressing its potential biases, calibration, and data governance. The authors’ warning regarding the legal implications of uploading unpublished or sensitive clinical data to third-party AI platforms is particularly salient. As we push toward integrating AI models into clinical workflows, establishing secure, localized processing environments will be as important as the algorithmic advancements themselves.

Ultimately, the successful integration of GenAI into both HTA and direct clinical practice depends on our ability to enforce rigorous evaluation standards while maintaining explicit human accountability for final decision-making. We commend the authors for providing a pragmatic, risk-based roadmap that helps bridge the gap between technical AI capabilities and methodological responsibility in healthcare.

## Data Availability

Data sharing is not applicable to this article as no new data were created or analyzed in this study.
